# InAs-mediated growth of vertical InSb nanowires on Si substrates

**DOI:** 10.1186/1556-276X-8-333

**Published:** 2013-07-24

**Authors:** Tianfeng Li, Lizhen Gao, Wen Lei, Lijun Guo, Huayong Pan, Tao Yang, Yonghai Chen, Zhanguo Wang

**Affiliations:** 1 Department of Physics, School of Physics and Electronics, Henan University, Kaifeng 475004, People's Republic of China; 2 Key Laboratory of Semiconductor Material Science, Institute of semiconductors, Chinese Academy of Science, Beijing 100083, People's Republic of China; 3 School of Electrical, Electronic and Computer Engineering, The University of Western Australia, 35 Stirling Hwy, Crawley 6009, Australia; 4 Key Laboratory for the Physics and Chemistry of Nanodevices, and Department of Electronics, Peking University, Beijing 100871, China

**Keywords:** Catalyst-free, MOCVD, InSb, Nanowire, Electron microscopy

## Abstract

In this work, InSb nanowires are grown vertically on Si (111) with metal organic chemical vapor deposition using InAs as seed layer, instead of external metal catalyst. Two groups of InSb nanowires are fabricated and characterized: one group presents Indium droplets at the nanowire's free end, while the other, in contrast, ends without Indium droplet but with pyramid-shaped InSb. The indium-droplet-ended nanowires are longer than the other group of nanowires. For both groups of InSb nanowires, InAs layers play an important role in their formation by serving as a template for growing InSb nanowires. The results presented in this work suggest a useful approach to grow catalyst-free InSb nanowires on Si substrates, which is significant for their device applications.

## Background

Indium antimonide (InSb), a kind of III-V semiconductor with a narrow bandgap (0.17 eV), a large bulk electron mobility (≈7.7×10^4^ cm^2^/V/s) [1], and a high thermoelectric figure of merit (0.6) [2], has been an attractive material for various applications such as high-speed and low-power electronics, infrared optoelectronics, quantum-transport studies, and thermoelectric power generation [3-5]. The heteroepitaxial growth of InSb films on Si surface has attracted much attention due to the potential of integrating InSb devices on Si substrate. However, because of the large lattice mismatch between InSb films and Si substrate (approximately 19.3%) [6], it is difficult to directly grow InSb film heteroepitaxially on Si substrate without generating defects.

Nanowires (NWs) are kinds of materials with a size in the range of nanometers. The lattice mismatch/strain in NWs is one of the most important features of NWs, in which the lattice mismatch/strain can be significantly relaxed due to their high surface/volume ratio and small lateral size, providing an opportunity to integrate InSb materials and devices on Si platform. It should be noted that gold, the most used seed particles for NW growth, is known to create detrimental midgap defects in silicon and should therefore be avoided in Si-compatible technological processes. So far, though some work has been devoted to external metal catalyst-free growth of InAs and GaAs NWs on Si [7-9], very few information is available on external metal catalyst-free growth of InSb NWs on silicon. In this work, we investigate the external metal catalyst-free growth of InSb NWs on Si substrates. Our results show that it is hard to grow InSb NWs directly on Si. However, using InAs as seeding layer, vertical InSb NWs can be readily achieved on Si substrates. The structural characteristics of InSb NWs are systematically studied and their underlying growth mechanisms are discussed as well.

## Methods

Vertical InSb NWs were grown on n-type Si (111) substrates in a close-coupled showerhead metal-organic chemical vapor deposition (MOCVD) system (Thomas Swan Scientific Equipment, Ltd., Cambridge, England) at a pressure of 100 Torr. Trimethylindium (TMIn), trimethylantimony (TMSb), and AsH_3_ were used as precursors and ultra-high purity H_2_ as carrier gas. Before being loaded into the growth chamber, Si substrates were first cleaned (ultrasonicated in trichloroethylene, acetone, isopropanol, and deionized water, sequentially), and etched in buffered oxide etch solution (BOE, six parts 40% NH_4_F and one part 49% HF) for 30 s to remove the native oxide, then rinsed in deionized water for 15 s and dried with N_2_. After that, the substrates were loaded into the MOCVD reactor chamber for NW growth. The InSb NWs were grown using the following layer sequence: first, InAs seed layer was grown at 550°C by simultaneously introducing TMIn (2×10^−6^ mole/min) and AsH_3_ (2×10^−4^ mole/min) into the reactor chamber for 2 min, as reported in previous work [10,11]; then, the growth temperature was reduced to 440°C with only AsH_3_ flowing. After that, the AsH_3_ flow was removed from the chamber, while TMSb (6.75×10^−5^) and TMIn (4.5×10^−6^) flows were simultaneously introduced into the reactor chamber to initiate the growth of InSb NWs. The InSb NWs were grown for 40 min and then cooled down with the protection of only hydrogen flow (TMIn and TMSb flows were removed from the reactor chamber during cooling). For comparison, InSb layers were also grown directly on Si (111) under the same growth conditions but without InAs seed layer. The morphology of InSb structures was characterized with field-emission scanning electron microscopy (FE-SEM; JSM-6700 F, JEOL, Akishima-shi, Japan)and transmission electron microscopy (TEM; Tecnai G20, 200 keV, FEI, Hillsboro, OR, USA). Raman scattering measurements were performed in a backscattering geometry at room temperature with a Jobin Yvon HR800 confocal micro-Raman spectrometer (HORIBA, Kyoto, Japan), in which a 514.5-nm line of an Ar-ion laser was used as the excitation source with the focus size around 1 μm and excitation power of 0.5 mW.

## Results and discussion

**Figure 1:**
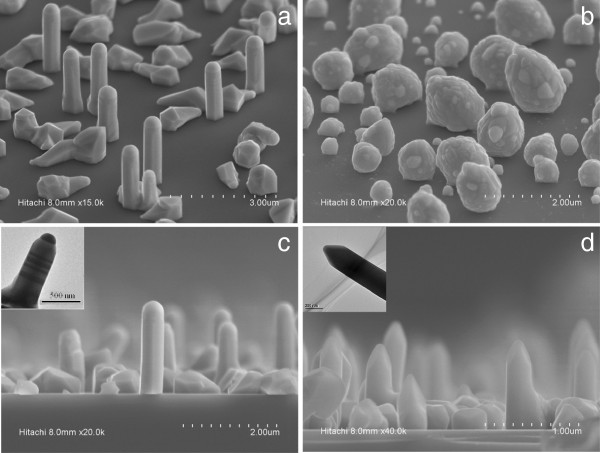
**SEM images of InSb NWs grown on Si substrate.** SEM image of the InSb NWs grown with **(a)** and without **(b)** InAs-seed-layer (tilt 45°); **(c)** side view of the InSb NWs showing a clear metallic droplet on their top. The inset (scale bar nm) shows the details on the final end of them (TEM); **(d)** the obtained InSb NWs without metallic droplet on the top. The inset shows details of this kind of NW (TEM).

Figure 1 shows the SEM images of InSb structures with and without InAs seed layer. Clearly, InSb NWs are formed in the sample with InAs seed layer, while no InSb NWs are observed in the sample without InAs seed layer. For the latter case, as shown in Figure 1b, only particle-like morphology is observed, instead of NWs. This indicates that InAs seed layer plays an important role in growing InSb NWs. Epitaxial growth of InSb is not trivial due to its large lattice constant (*a*_0_ = 0.648 nm) compared to other III-V-semiconductor materials. As reported in our previous work [11], vertical InAs NWs can be directly heteroepitaxially grown on Si substrates at about 550°C (Additional file 1: Figure S1 and Additional file 2: Figure S2). Therefore, the InAs seed layer deposited at 550°C can form InAs NWs, which provide a template for the subsequent growth of InSb NWs. With the growth temperature being reduced to 440°C, TMIn and TMSb are introduced into the reactor chamber, and the InSb growth is initiated on the template provided by the InAs seed layer, which facilitates the formation of InSb NWs. This growth mechanism is confirmed by the chemical composition distribution along the InSb NWs, which will be discussed later. It should be noted that the parasitic growth of non-wire-like InSb material is also observed in the form of InSb structures with non-well-developed crystal faces [12]. All vertical InSb NWs are grown along the (111) direction perpendicular to Si substrate, as shown in Figure 1a.

Figure 1c,d shows the side view SEM images of InSb NWs obtained with InAs seed layer. Two groups of NWs are observed on the sample surface. The first group (as shown in Figure 1c) clearly shows a droplet-like end at the NW top. These NWs are about 2 μm in length, and 200 to 300 nm in diameter. Combined with the inset of Figure 1c, it is observed that the indium droplet on the NW top shows an identical (or slightly smaller) diameter to that of InSb NWs, which is a typical phenomenon for NWs grown with the vapor–liquid-solid (VLS) growth model and has also been observed in InSb NWs grown on InAs substrates [12]. The second group of InSb NWs (as shown in Figure 1d), however, do not present droplet-like end at the NW top, and these NWs present a little small length (about 1 μm), but a similar sectional diameter to that of the first group. These two groups of NWs are observed in different areas of the sample surface.

**Figure 2:**
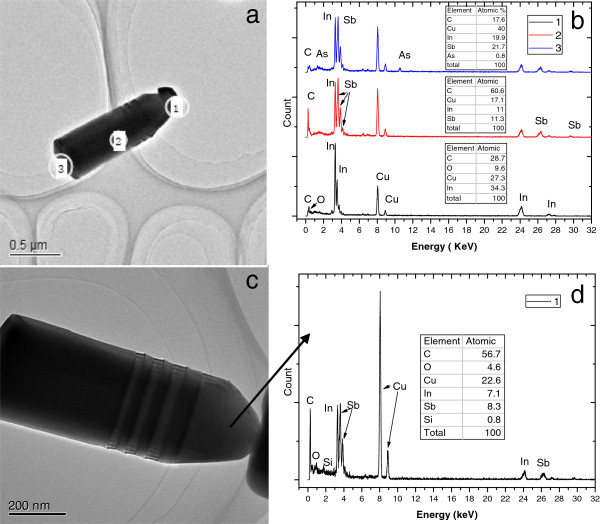
**TEM image and the EDS spectra of an InSb NW. (a)** TEM image of an InSb NW terminating with an indium droplet. The ‘1’, ‘2’, and ‘3’ circles indicate the regions where the EDS spectra shown in **(b)** are presented, respectively. **(c)** TEM image of a NW without a droplet on its end. The arrow indicates the region where the EDS spectra shown in **(d)** are acquired.

In order to probe the chemical composition distribution in the NWs, energy dispersive spectroscopy (EDS) measurements are performed on several NWs of both groups, where the EDS spectra are obtained using a TEM electron beam operated at 200 keV. Figure 2a presents the TEM image of a NW with a droplet-like end. The framed regions ‘1’ , ‘2’ , and ‘3’ drawn on the NW TEM image indicate the areas from which the EDS spectra are taken. The EDS spectra measured in regions 1, 2, and 3 are presented in Figure 2b. The ‘1’ of Figure 2b shows the EDS spectrum obtained on the NW top with the inset showing the chemical composition. The spectrum is composed of two main peaks corresponding to indium and copper (coming from copper grid). The ‘2’ of Figure 2b (obtained in the body area) show two main peaks corresponding to indium and antimony. The inset of Figure 2b indicates that the chemical composition of indium and antimony are almost equal. These results confirm that the rod body is dominated by InSb materials, while the top end is dominated by the indium particle. The EDS spectrum taken at the bottom of the NW is shown as ‘3’ in Figure 2b. In addition to indium and antimony, arsenic signal is also clearly observed although it is much weaker compared with indium and antimony signals. This can be interpreted that the arsenic signal arises from the InAs seed layer which might be wrapped up by InSb shell layers. A schematic illustration of InSb NW with indium droplet on its top is shown in Additional file 2: Figure S2b, where the InSb NWs are formed via the VLS model. In this growth model, excess indium forms on the side face and top surface of InAs NWs at some regions before the deposition of InSb due to As extravasation after switching off AsH_3_ flow. When InSb layer is deposited, InSb is incorporated onto the side face and top surface of InAs NWs, leading to the initiation of InSb NWs. During the growth of InSb NWs, indium droplet on the NW top induces a faster axial growth compared with the lateral shell growth. Such growth process leads to the formation of InSb NWs with larger diameter (core-shell structure), which is confirmed by the larger diameter of InSb wires (approximately 200 nm) observed here in contrast to the small diameter (approximately 70 nm) of InAs NWs shown in Additional file 2: Figure S2a (grown under the same growth condition as the InAs seed layers). The faster axial growth in InSb NWs is well supported by the absence of arsenic signal in the EDS spectra of body part of InSb NWs and the presence of arsenic signal in the EDS spectra of the bottom part of InSb NWs.

A similar analysis is performed on the other group of NWs without droplet-like ends, where the TEM image and the related EDS spectra are shown in Figure 2c. Note the EDS spectra are obtained in the area indicated by the arrow in Figure 2c. The EDS spectra measured on the free end of InSb NW shows the same stoichiometry as the NW body with InSb. Similarly, arsenic signal is also observed at the bottom of InSb NW (composition spectra not shown here). This indicates that except the indium droplet end, the second group of NWs shows a similar chemical composition distribution to the first group of NWs. The absence of In droplets on the NW top end might be related to the catalyst self-consumption during the growth, which has been observed in other catalyst-assisted NWs [13]. Such catalyst self-consumption during the NW growth will lead to a smaller axial growth rate for the NWs [12,14], which is confirmed by the relatively small length of the second group of NWs. All the second group of InSb NWs (without In droplet on the top end) present a length less than 1 μm, while the first group of InSb NWs (with indium droplet on the top end) are all longer than 2 μm. It should be noted that that catalyst self-consumption during the NW growth will lead to the formation of randomly located NWs with wide distributed lengths, which, however, does not agree with the morphology observed for the second group of InSb NWs. As shown in Figure 1, the second group of NWs has a narrow length size distribution and is homogeneously located in well-defined parts of the substrate surface, which does not accord with the catalyst consumption dependence on the catalyst dimension. This suggests that the growth process of the second group of InSb NWs is more complicated compared with that of the first group InSb NWs, and some other factors except VLS model might take effect. Although it is difficult to present a complete growth mechanism for the second group of InSb NWs, the important role of InAs seed layer is clearly evidenced, which serves as a template for initiating the growth of InSb NWs.

**Figure 3:**
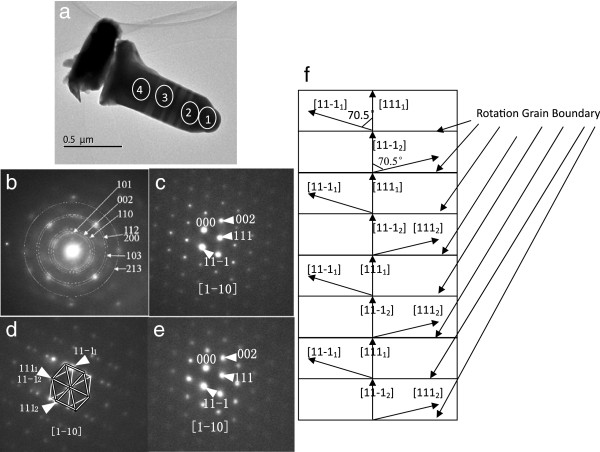
**TEM image and SAED pattern of an InSb NW. (a)** TEM image of an InSb nanowire terminating with a droplet; **(b)** SAED pattern from the droplet shown in the area 1 of (a); **(c)** SAED pattern from area 2 shown in (a); **(d)** SAED pattern from area 3 shown in (a); **(e)** SAED pattern from area 4 shown in (a). **(f)** Structural diagram of rotation grain boundary.

To obtain a deep insight into the lattice characteristics of the NWs, TEM imaging were performed along the [−110] zone axis (cubic notation). Figure 3a shows the TEM image of a representative NW of the first group (with indium droplet top ends). The regions ‘1’ , ‘2’ , ‘3’ , and ‘4’ indicate the regions where the selected-area electron diffraction (SAED) analysis is performed. Note that region ‘1’ is on the indium droplet end, while regions ‘2’ , ‘3’ , and ‘4’ are on the NW body. The SAED spectrum measured at ‘1’ , ‘2’ , ‘3’ , and ‘4’ is shown in Figure 3b,c,d,e, respectively. It can be observed that the indium droplet shows poly-crystalline structures (metal) (see Figure 3b), while the NW body just below the indium droplet present zinc blende structure (InSb semiconductor) (see Figure 3c), which is consistent with previous results reported [15-17] for Au or Ag-catalyzed InSb NWs. The SAED pattern from area 3 (Figure 3d) shows two sets of diffraction patterns [18], and both of them are [1-10] zone axis diffraction patterns. One pattern indexed by 1 presents a relative 70.5° rotation with respect to the other pattern indexed by 2. 111_1_ coincides with 11-1_2,_ and two patterns reveal the same {111} plane class parallel to growth direction of NW. Figure 3f presents the structural diagram of rotation grain boundary. In Figure 3a, the dark contrast area represents the [1-10] orientation indexed by 1, while the bright contrast area represents the [1-10] orientation indexed by 2. The interfaces between bright areas and dark areas indicate the rotation grain boundaries. There are eight rotation grain boundaries in InSb NW as shown in Figure 3a. The SAED pattern from area 4 is shown in Figure 3e, which shows a cubic zinc blende, the same structure as that shown in Figure 3c. The second group of InSb NWs (without indium droplet top ends) demonstrates the same lattice structure as the first group InSb NWs with indium droplet top ends (SAED results are shown Additional file 3: Figure S3).

**Figure 4:**
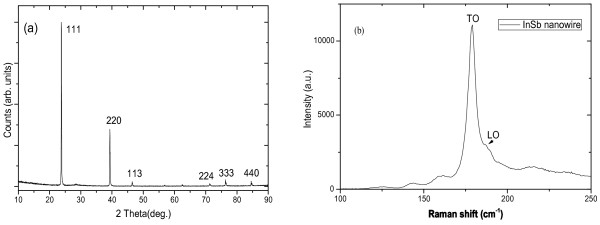
**XRD and Raman spectroscopy of InSb NWs. (a)** X-ray diffraction scan of a selected InSb NWs array sample, confirming the epitaxial relationship between InAs (111) and Si (111) substrate; **(b)** Raman spectroscopy measurements on InSb NWs grown on Si substrate.

Figure 4a shows a typical longitudinal 2θ-ω XRD scan measured from InSb ensemble NW sample. The peaks at 23.8° and 76.3° arise from the 111 and 333 reflections of zinc-blende-structured InSb, respectively [12]. All the observed InSb reflections match those of Si (111), indicating the epitaxial growth of InSb NWs facilitate perpendicular to the Si substrate. It should be mentioned that a portion of XRD signal might originate from the InSb particles deposited on the substrate surface. Figure 4b shows the Raman spectrum of InSb ensemble NW sample. It is observed that the Raman spectrum is dominated by a peak centered at 179/cm, which can be ascribed to the transverse-optical phonon mode of InSb, as reported in InSb NWs grown on Si/SiO_2_[19]. Beside this main peak, a shoulder located at 190/cm is also observed, which is assigned to longitudinal-optical phonon mode of InSb. These XRD and Raman results further support and confirm the formation of InSb NWs in our work.

## Conclusions

In conclusion, InSb NWs have been grown on Si substrates using an InAs seed layer instead of external metal catalyst. The deposition of InAs seed layer leads to the growth of InAs NWs, which serve as a template for the subsequent initiation and growth of InSb NWs. Two different groups of InSb NWs are observed: one with indium droplet top end and the other without indium droplet top end. Though the growth of the first group of InSb NWs is evidenced to follow VLS mode, the growth of the second group of InSb NWs is more complex, the complete picture of which is not clear yet. Despite this, the work demonstrates a method towards the realization of Au catalyst-free InSb NWs, which is important for their ultimate device applications.

## Abbreviations

NWs: Nanowires; MOCVD: Metal-organic chemical vapor deposition; SAED: Selected-area electron diffraction; EDS: Energy dispersive spectroscopy; SEM: Scanning electron microscopy.

## Competing interests

The authors declare that they have no competing interests.

## Authors’ contributions

TFL carried out the experiments, data analysis, and prepared the manuscript. WL and LZG contributed to the data collection and the experimental analysis. TY, ZGW, and HYP took part in the discussion and coordination. YHC and LJG designed the experiments, analyzed the data, and modified the manuscript. All authors read and approved the final manuscript.

## Supplementary Material

Additional file 1: Figure S1 FE-SEM (450° tilted view) of InAs nanowires grown for 7 min on Si (111) substrates at 550°C. Click here for file

Additional file 2: Figure S2 FE-SEM image of InAs nanowires and schematic illustration of InSb nanowire. **(a)** FE-SEM (45° tilted view) of the InAs nanowires grown for 2 min on Si (111) substrates at 550°C. **(b)** Schematic illustration of InSb nanowire with indium droplet on Si (111) substrate. Click here for file

Additional file 3: Figure S3 TEM image and SAED pattern of an InSb NW with crystalline InSb tip. **(a)** TEM image of the topmost part of a nanorod with crystalline InSb tip. The SAEDs of the image in the tip **(b)** and in the rod body **(c**,**d)** are also shown. (b, c, and d) correspond to cubic regions with alternate orientation due to twinning. The twinning is pointed out by the bright and dark stripes that correspond to different regions with opposite orientations of the crystal. Click here for file

## References

[B1] Riikonen J Tuomi T Lankinen A Sormunen J Saynatjoki A Knuuttila L Lipsanen H McNally PJ O’Reilly L Danilewsky A Sipila H Vaijarvi S Lumb D Owens A Synchrotron X-ray topography study of defects in indium antimonide P-I-N structures grown by metal organic vapour phase epitaxy J Mater Sci Mater Electron 2005 8 449 10.1007/s10854-005-2313-5

[B2] Seol JH Moore AL Saha SK Zhou F Shi L Ye QL Scheffler R Mingo N Yamada T Measurement and analysis of thermopower and electrical conductivity of an indium antimonide nanowire from a vapor–liquid-solid method J Appl Phys 2007 8 023706 10.1063/1.2430508

[B3] Mourik V Zuo K Frolov SM Plissard SR Bakers EPAM Kouwenhoven LP Signatures of majorana fermions in hybrid superconductor semiconductor nanowire devices Science 2012 8 1003 10.1126/science.1222360 22499805

[B4] Nilsson HA Caroff P Thelander C Larsson M Wagner JB Wernersson LE Samuelson L Xu HQ Giant, level-dependent g factors in InSb nanowire quantum dots Nano Lett 2009 8 3151 10.1021/nl901333a 19736971

[B5] Nilsson HA Caroff P Thelander C Lind E Karlstrom O Wernersson LE Temperature dependent properties of InSb and InAs nanowire field-effect transistors Appl Phys Lett 2010 8 153505 10.1063/1.3402760

[B6] Murata K Ahmad NB Tamura Y Mori M Tatsuyama C Tambo T Low-temperature growth of InSb(111) on Si (111) substrate J Cryst Growth 2007 8 203

[B7] Tomioka K Motohisa J Hara S Fukui T Control of InAs nanowire growth directions on Si Nano Lett 2008 8 3475 10.1021/nl802398j 18783279

[B8] Paek JH Nishiwaki T Yamaguchi M Sawaki N Catalyst free MBE-VLS growth of GaAs nanowires on (111)Si substrate Phys Status Solidi C 2009 8 1436 10.1002/pssc.200881520

[B9] Plissard S Dick KA Wallart X Caroff P Gold-free GaAs/GaAsSb heterostructure nanowires grown on silicon Appl Phys Lett 2010 8 121901 10.1063/1.3367746

[B10] Wei W Bao XY Soci C Ding Y Wang ZL Wang DL Direct heteroepitaxy of vertical InAs nanowires on Si substrates for broad band photovoltaics and photodetection Nano Lett 2009 8 2926 10.1021/nl901270n 19624100

[B11] Li TF Chen YH Lei W Zhou XL Luo S Hu YZ Wang LJ Yang T Wang ZG Effect of growth temperature on the morphology and phonon properties of InAs nanwires on Si substrates Nanoscale Res Lett 2011 8 463 10.1186/1556-276X-6-463 21777417 PMC3211884

[B12] Mandl B Dick KA Kriegner D Keplinger M Bauer G Stangl J Deppert K Crystal structure control in Au-free self-seeded InSb wire growth Nanotechnology 2011 8 145603 10.1088/0957-4484/22/14/145603 21346304

[B13] Cao L Garipean B Atchison JS Ni C Nabet B Spanier JE Instability and transport of metal catalyst in the growth of tapered silicon nanowires Nano Lett 1852 8 6 10.1021/nl060533r 16967990

[B14] Pozuelo M Zhou H Lin S Lipman SA Goorsky MS Hicks RF Kodambaka S Self-catalyzed growth of InP/InSb axial nanowire heterostructures J Crys Grow 2011 8 6 10.1016/j.jcrysgro.2011.06.034

[B15] Caroff P Wagner JB Dick KA Nilsson HA Jeppsson M Deppert K Samuelson L Wallenberg LR Wernersson LE High-quality InAs/InSb nanowire heterostructures grown by metal-organic vapor-phase epitaxy Small 2008 8 878 18576282 10.1002/smll.200700892

[B16] Caroff P Messing ME Borg BM Dick KA Deppert K Wernersson LE InSb heterostructure nanowires: MOVPE growth under extreme lattice mismatch Nanotechnology 2009 8 495606 10.1088/0957-4484/20/49/495606 19904026

[B17] Vogel AT Boor J Becker M Wittemann JV Mensah SL Werner P Schmidt V Ag-assisted CBE growth of ordered InSb nanowire arrays Nanotechnology 2011 8 015605 10.1088/0957-4484/22/1/015605 21135461

[B18] Vogel AT Boor J Becker M Wittemann JV Mensah SL Werner P Schmidt V Fabrication of high-quality InSb nanowire arrays by chemical beam epitaxy Cryst Growth Des 1896 8 11

[B19] Wang Y Chi J Banerjee K Grutzmacher D Schapers T Lu JG Field effect transistor based on single crystalline InSb nanowire J Mater Chem 2011 8 2459 10.1039/c0jm03855e

