# Supplementary material for: InAs-mediated growth of vertical InSb nanowires on Si substrates
Source: Nanoscale Res Lett. 2013 Jul 24;8(1):333. doi: 10.1186/1556-276X-8-333 (PMC3726463; doi:10.1186/1556-276X-8-333)
Supplement: Additional file 1: Figure S1 — FE-SEM (450° tilted view) of InAs nanowires grown for 7 min on Si (111) substrates at 550°C. [file 1556-276X-8-333-S1.pdf]

Supporting Information

Figure S1

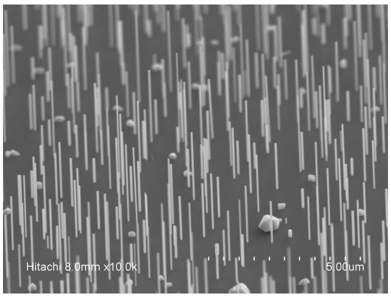

Figure S1 FE-SEM（45^0 tilted view）of the InAs nanowires grown for 7 min on Si(111) substrates grown at grown at 550 ^{0}C .
