# Supplementary material for: InAs-mediated growth of vertical InSb nanowires on Si substrates
Source: Nanoscale Res Lett. 2013 Jul 24;8(1):333. doi: 10.1186/1556-276X-8-333 (PMC3726463; doi:10.1186/1556-276X-8-333)
Supplement: Additional file 2: Figure S2 — FE-SEM image of InAs nanowires and schematic illustration of InSb nanowire. (a) FE-SEM (45° tilted view) of the InAs nanowires grown for 2 min on Si (111) substrates at 550°C. (b) Schematic illustration of InSb nanowire with indium droplet on Si (111) substrate. [file 1556-276X-8-333-S2.pdf]

Supporting Information

Figure S2

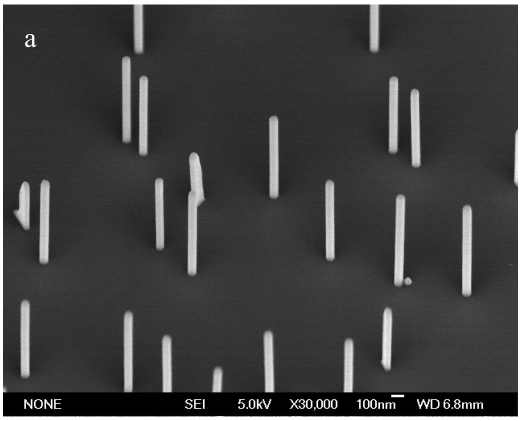

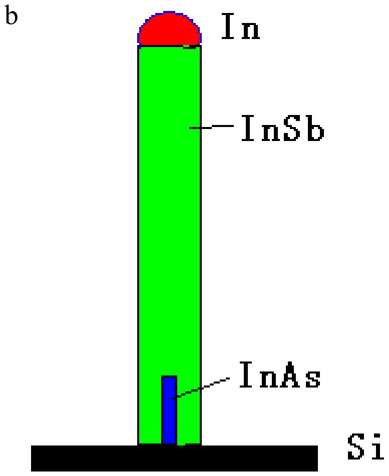

Figure S2 FE-SEM image of the InAs nanowires and schematic illustration of InSb nanowire.

(a) FE-SEM (45^0 tilted view) of the InAs nanowires grown for 2 min on Si (111) substrates at 550 °C. (b) Schematic illustration of InSb nanowire with Indium droplet on Si (111) substrate.
