# Supplementary material for: InAs-mediated growth of vertical InSb nanowires on Si substrates
Source: Nanoscale Res Lett. 2013 Jul 24;8(1):333. doi: 10.1186/1556-276X-8-333 (PMC3726463; doi:10.1186/1556-276X-8-333)
Supplement: Additional file 3: Figure S3 — TEM image and SAED pattern of an InSb NW with crystalline InSb tip. (a) TEM image of the topmost part of a nanorod with crystalline InSb tip. The SAEDs of the image in the tip (b) and in the rod body (c,d) are also shown. (b, c, and d) correspond to cubic regions with alternate orientation due to twinning. The twinning is pointed out by the bright and dark stripes that correspond to different regions with opposite orientations of the crystal. [file 1556-276X-8-333-S3.pdf]

Supporting Information

Figure S3

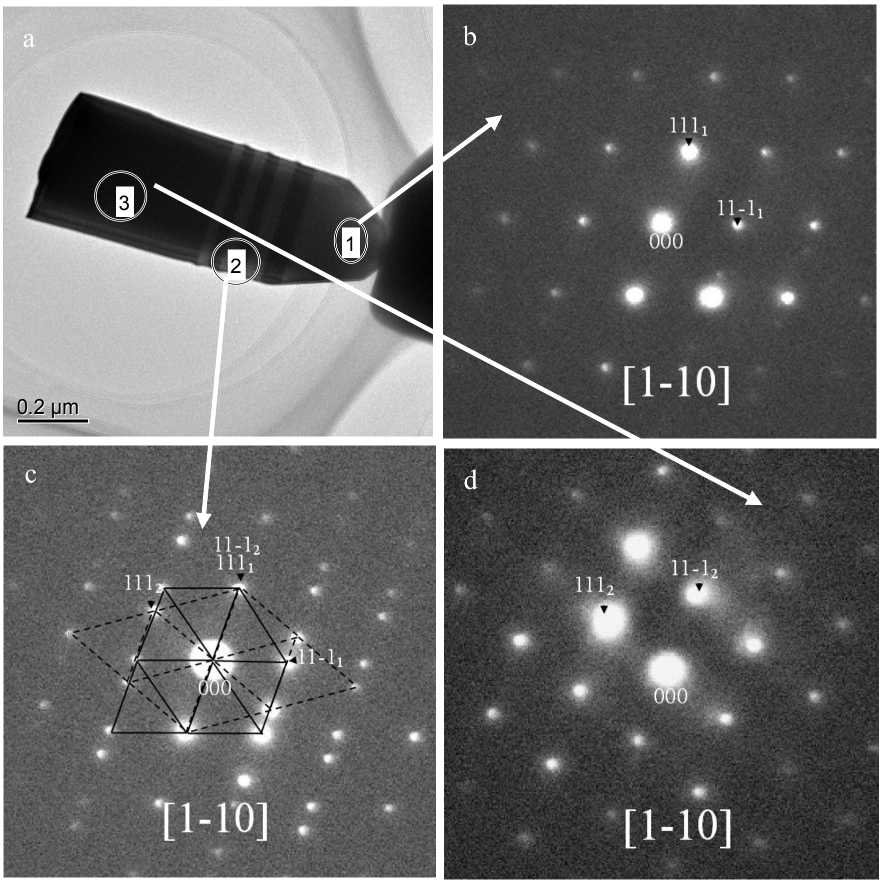

Figure S3. TEM image and the SAED pattern of an InSb NW with crystalline InSb tip. (a) TEM image of the topmost part of a nanorod with crystalline InSb tip. The SAEDs of the image in the tip (b) and in the rod body ((c), (d)) are also shown. Figure (b), (c) and (d) correspond to cubic regions with alternate orientation due to twinning. The twinning is pointed out by the bright and dark stripes that correspond to different regions with opposite orientations of the crystal.
